# Is Quality and Completeness of Reporting of Systematic Reviews and Meta-Analyses Published in High Impact Radiology Journals Associated with Citation Rates?

**DOI:** 10.1371/journal.pone.0119892

**Published:** 2015-03-16

**Authors:** Christian B. van der Pol, Matthew D. F. McInnes, William Petrcich, Adam S. Tunis, Ramez Hanna

**Affiliations:** 1 Department of Radiology, University of Ottawa, Ottawa, Ontario, Canada; 2 Clinical Epidemiology Program, Ottawa Hospital Research Institute, Ottawa, Ontario, Canada; University of Glasgow, UNITED KINGDOM

## Abstract

**Purpose:**

The purpose of this study is to determine whether study quality and completeness of reporting of systematic reviews (SR) and meta-analyses (MA) published in high impact factor (IF) radiology journals is associated with citation rates.

**Methods:**

All SR and MA published in English between Jan 2007–Dec 2011, in radiology journals with an IF >2.75, were identified on Ovid MEDLINE. The Assessing the Methodologic Quality of Systematic Reviews (AMSTAR) checklist for study quality, and the Preferred Reporting Items for Systematic Reviews and Meta-Analyses (PRISMA) checklist for study completeness, was applied to each SR & MA. Each SR & MA was then searched in Google Scholar to yield a citation rate. Spearman correlation coefficients were used to assess the relationship between AMSTAR and PRISMA results with citation rate. Multivariate analyses were performed to account for the effect of journal IF and journal 5-year IF on correlation with citation rate. Values were reported as medians with interquartile range (IQR) provided.

**Results:**

129 studies from 11 journals were included (50 SR and 79 MA). Median AMSTAR result was 8.0/11 (IQR: 5–9) and median PRISMA result was 23.0/27 (IQR: 21–25). The median citation rate for SR & MA was 0.73 citations/month post-publication (IQR: 0.40–1.17). There was a positive correlation between both AMSTAR and PRISMA results and SR & MA citation rate; ρ=0.323 (*P*=0.0002) and ρ=0.327 (*P*=0.0002) respectively. Positive correlation persisted for AMSTAR and PRISMA results after journal IF was partialed out; ρ=0.243 (*P*=0.006) and ρ=0.256 (*P*=0.004), and after journal 5-year IF was partialed out; ρ=0.235 (*P*=0.008) and ρ=0.243 (*P*=0.006) respectively.

**Conclusion:**

There is a positive correlation between the quality and the completeness of a reported SR or MA with citation rate which persists when adjusted for journal IF and journal 5-year IF.

## Introduction

Impact factor (IF) is a metric that attempts to quantify the overall citation rate of a journal [1]. It is widely considered a measure of journal prestige, and is often used to measure the research performance of investigators and institutions [2–4]. As a journal’s IF depends on the number of times a journal’s articles are cited, there has been interest across a variety of medical specialties to determine factors which are associated with citation [5–9]. More recently, there has been increased reference to journal IF in radiology publications [10–13].

A journal’s IF is calculated by determining the number of times the articles published in a journal over a preceding period of time are cited by indexed journals within a year, divided by the total number of “citable items” published in the journal during the same preceding period of time [14]. This calculation is often skewed by outlying articles, specifically articles that receive a high number of post-publication citations [10,15]. A journal’s IF therefore does not represent the number of citations for each individual article, but rather the sum of all citations of all published articles. The impact of any single article cannot be assumed based on the IF of the journal it was published in [16,17].

As one of the highest levels of evidence available in the diagnostic imaging literature, systematic reviews (SR) and meta-analyses (MA) are conducted in attempt to produce high impact findings [18]. These pool existing data to eliminate bias, increase sample size and ultimately provide stronger answers to clinical questions than can be achieved from any individual component study [19–21]. Yet factors associated with post-publication citations of diagnostic imaging related SR & MA have not been well characterized. In particular, it is unclear if the quality or completeness of SR & MA affects how often they are cited.

Several tools have been developed to quantify the quality and completeness of SR & MA [22–24]. These include “The Assessing the Methodologic Quality of Systematic Reviews” (AMSTAR) [22] to assess quality, and the “Preferred Reporting Items for Systematic Reviews and Meta-Analyses” (PRISMA) statement to assess completeness of reporting [24]. The purpose of this study is to determine whether study quality and completeness of reporting of SR & MA published in high IF radiology journals are associated with individual article citation rates.

## Materials and Methods

### Study selection and data extraction

A search was performed in MEDLINE to identify SR & MA published in radiology journals with an impact factor >2.75 based on the Thomson ISI ranking. A radiology journal was defined as any journal included on the Thomson ISI ranking that primarily published articles related to any aspect of medical imaging. This included any radiology subspecialty specific journals. Medical imaging related SR & MA published in non-radiology journals were excluded. A threshold of 2.75 was chosen to include studies in the most frequently cited radiology journals while limiting the total number of studies to a manageable amount. The search was limited to English language articles published between Jan 2007–Dec 2011. Two investigators independently retrieved and reviewed all included articles, with discrepancies resolved by consensus (A.S.T., a third year radiology resident and M.D.F.M., a staff radiologist with more than 3 years of experience in the performance and review of SR & MA).

Data extraction was performed independently on included articles by two investigators (A.S.T. and R.H., both third year radiology residents) and assessed using AMSTAR & PRISMA checklists (S1 Fig. and S1 Table). The first ten articles were reviewed in consensus to become familiar with application of the AMSTAR and PRISMA checklists. Following this, all remaining articles were reviewed independently. Discrepancies were resolved through consensus or, if there was persistent disagreement, discussed with a third investigator (M.D.F.M.). The detailed methods can be found in the previously published paper titled “Association of study quality with completeness of reporting: have completeness of reporting and quality of systematic reviews and meta-analyses in major radiology journals changed since publication of the PRISMA statement?” [25].

### Outcome measure assessment

The number of citations for each individual SR & MA was documented based on the number of citations indexed through Google Scholar [26] as of April 5, 2014. A post-publication citation *rate* was then calculated by dividing the total number of citations for each article by the total number of months since the earliest date of publication (e.g. epub ahead of print date) [27,28]. The purpose of using a citation rate rather than absolute post-publication citation counts was to eliminate the effect of varying amounts of time since publication, since studies published earlier have had more time to accumulate citations.

### Statistical analysis

Median AMSTAR and PRISMA results were reported, along with the interobserver agreement as calculated using the kappa coefficient (κ) for all SR & MA except the first ten, which were reviewed in consensus. Spearman correlation coefficients (ρ) were used to assess for correlation between AMSTAR result and citation rate, PRISMA result and citation rate, journal IF and citation rate, and journal 5-year IF and citation rate. A multivariate analysis was performed: Spearman partial correlations were performed to assess the associations between AMSTAR or PRISMA results and post-publication citation rate while controlling for the effect of journal IF and journal 5-year IF. Scatter plots were created to demonstrate the distribution of citation rates relative to AMSTAR and PRISMA results with polynomial lines fitted to data using LOESS (local polynomial regression fitting) [29]. All statistical analysis was performed using SAS version 9.2 (SAS Institute Inc. Cary, NC, USA). Values are reported as medians with interquartile range (IQR).

## Results

**Fig 1:**
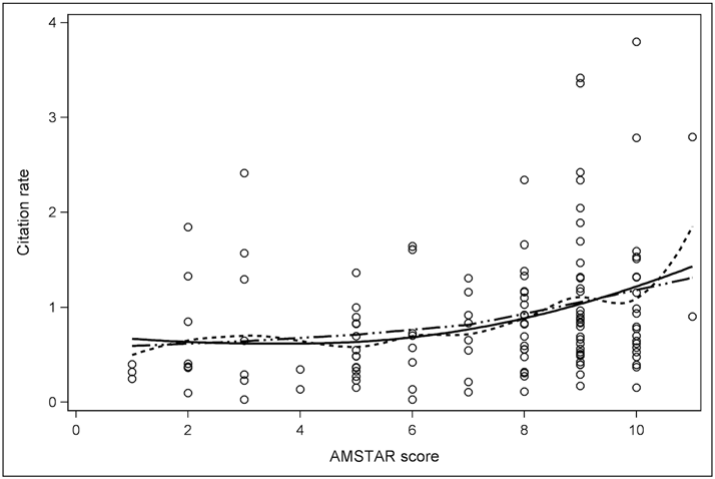
Scatter plot demonstrating the distribution of citations rates for each AMSTAR result. Solid line: polynomials fitted to all data using LOESS [29]. Dashed line: polynomials linking subsets of data. Double dot-and-dash line: straight lines fitted to subsets of data.

**Fig 2:**
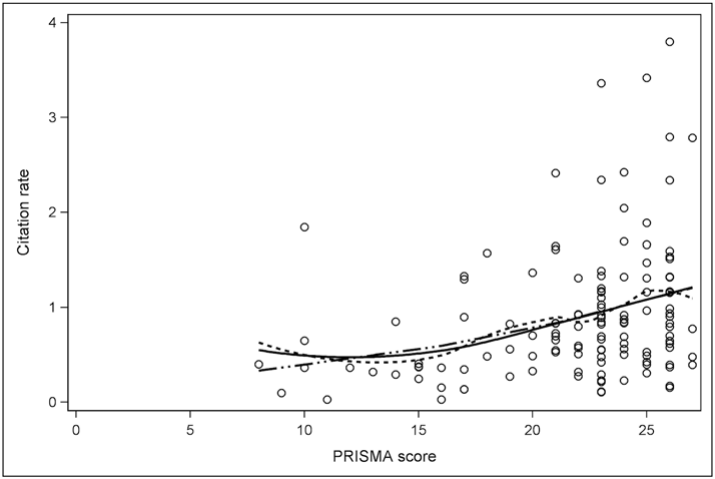
Scatter plot demonstrating the distribution of citations rates for each PRISMA result. Solid line: polynomials fitted to all data using LOESS [29]. Dashed line: polynomials linking subsets of data. Double dot-and-dash line: straight lines fitted to subsets of data.

129 studies from 11 journals were identified that met our inclusion criteria [30–158] (S2 Table). A meta-regression analysis [159] included by Tunis et al. [25] was excluded from our study since it was not a systematic review. The median AMSTAR result was 8.0/11 (IQR: 5–9) and median PRISMA result was 23.0/27 (IQR: 21–25). The overall inter-observer agreement was moderate for the PRISMA results with κ = 0.57, and higher for the AMSTAR results with κ = 0.69. The median citation rate for SR & MA was 0.73 citations/month post-publication (IQR: 0.40–1.17). Scatter plots show the distribution of citation rates relative to AMSTAR result (Fig. 1) and PRISMA result (Fig. 2).

A positive correlation was observed between both AMSTAR and PRISMA results and SR & MA citation rates; ρ = 0.323 (*P* = 0.0002) and ρ = 0.327 (*P* = 0.0002) respectively. Slightly stronger correlation was observed between journal IF and citation rates; ρ = 0.333 (*P* = 0.0001), and journal 5-year IF and citation rates; ρ = 0.379 (*P*<0.0001).

After multivariate analysis, a positive correlation persisted for AMSTAR and PRISMA results after journal IF was partialed out; ρ = 0.243 (*P* = 0.006) and ρ = 0.256 (*P* = 0.004) respectively. Similarly, a positive correlation persisted after journal 5-year IF was partialed out; ρ = 0.235 (*P* = 0.008) and ρ = 0.243 (*P* = 0.006) respectively.

## Discussion

Our results suggest that the quality and completeness of SR & MA reported in high impact radiology journals is associated with post-publication citation rates. This association persisted on multivariate analysis. Although nearly the same study population was used as in a study by Tunis et al. [25], the purpose of our study was entirely different. Thus we felt an independent publication was warranted.

Radiology journals with an IF >2.75 may be more likely to publish SR & MA. Even though we limited our assessment to journals with a high IF, our use of multivariate analysis to partial out the effect of journal IF and 5-year IF should help correct for the fact that these journals are more frequently cited. Despite including only “high impact” journals, at the time of our analysis, none of the journals required submission of a PRISMA checklist along with a submitted manuscript. *Radiology* was the only journal that had endorsed the PRISMA statement. It is our hope that our findings encourage editors to endorse and authors to adhere to the PRISMA checklist in the future.

### Comparison to other studies

Prior studies have investigated factors associated with post-publication citation counts. Several factors shown to correlate with more citations include: study design and study topic in the urology literature [5]; high levels of evidence, large sample size, multi-institutional studies and conflict of interest disclosure in the orthopedic surgery literature [6]; extended description of statistical analysis [7]; statistically significant papers in the psychiatry literature [8]; being indexed in numerous databases, number of authors, clinical relevance scores and number of cited references [9]; and article length in the general medicine literature [160]. We are unaware of any prior work assessing the effect of SR & MA study quality and completeness on post-publication citation rates.

A study by Royle et al. investigated factors associated with higher citation counts of SR [16]. They found that the number of authors, first author from the United States, an ICD-10 chapter heading of Neoplasms, type of intervention classified as Investigation, Diagnostics or Screening, and having an international collaboration all correlated with increased citation counts. Similar to our study, they found that journal IF was a stronger predictor of citations.

A prior investigation of studies that were originally submitted to an emergency medicine specialty meeting found study design and quality did not correlate with post-publication citation rates [161]. Unlike in our study, they did not exclusively evaluate SR & MA, did not apply the AMSTAR and PRISMA checklists, and did not specifically look at the diagnostic imaging literature. Similar to our study and the study by Royle et al., they found that journal IF was more important than any other variable for post-publication counts.

It is interesting to note that our results confirm previous findings—namely that higher quality studies are cited more frequently. It is also interesting to note that the median number of citations/month was 0.73 (8.7 citations/year); this is considerably higher than the number of citations typically seen in our included cohort of radiology journals whose 2011 impact factors range from 2.75–6.07 [162]. This supports prior findings that studies of higher levels of evidence are cited more often [5,6,163].

### Limitations

There are several limitations to this study. Cross-referencing Google Scholar citation counts with other known citation databases such as Scopus [164] or the Thomson Reuters Web of Science [165] may have been beneficial since absolute citation counts have been shown to vary between databases [166]. However we were comparing relative citation counts between articles, and several studies have shown a strong correlation between Google Scholar citations counts and the Thomson Reuters Web of Science citation counts [167–169]. Furthermore, Google Scholar is arguably more comprehensive than other citation databases in certain fields [170,171]. Another limitation was that our calculation of citation rate, by dividing the total number of citations by time since publication, assumes that the citation frequency is independent of the time since publication. Given the difficulty in predicting the time course of citations, which can vary considerably over time between articles and depend on multiple factors including the article topic and the journal of publication, this could introduce a source of bias in our analysis [172–174]. However we felt that our calculated citation rate based on the number of citations over a three to seven year period following publication was adequate and that any bias is likely to apply evenly over all studies, thus minimizing the impact on our study conclusions. Furthermore, the interface of Google Scholar and other citation indexes do not allow for practical extraction of monthly citation information.

Several additional limitations outlined and addressed by Tunis et al. [25] are applicable to our study: the assessment of journals was not blinded to the journal or time of publication, our search was limited to radiology journals with high IF and thus does not represent the totality of the radiology literature, the selection of impact factor threshold was somewhat arbitrary (and was chosen as a practical means to result in a reasonable number of articles to review), and finally our interobserver agreement was only moderate. We believe that our moderate interobserver agreement was due to many items being flagged as unclear by one reviewer to be discussed with the other.

### Conclusion

In conclusion, there is positive correlation between the quality and the completeness of SR & MA published in high impact radiology journals with citation rate, which persists when adjusted for journal IF and journal 5-year IF. Although citation counts took on a wide range of values for a particular AMSTAR and PRISMA score, this study provides statistical evidence against there being “no relationship” with study quality and completeness and post-publication citations. This reinforces the importance of complete reporting and following publishing guidelines for authors of SR and MA, and might encourage more journals to endorse these guidelines.

## Supporting Information

S1 Fig PRISMA flowchart. (PDF)

S1 Table PRISMA checklist. (DOC)

S2 Table List of included articles with total PRISMA and AMSTAR results. (DOC)
