# Supplementary material for: Is Quality and Completeness of Reporting of Systematic Reviews and Meta-Analyses Published in High Impact Radiology Journals Associated with Citation Rates?
Source: PLoS One. 2015 Mar 16;10(3):e0119892. doi: 10.1371/journal.pone.0119892 (PMC4361663; doi:10.1371/journal.pone.0119892)
Supplement: S2 Table — (DOC) [file pone.0119892.s003.doc]

Table S2**.** List of included articles with total PRISMA and AMSTAR results.

| Reference | Journal | PRISMA | AMSTAR | Article Type |
| --- | --- | --- | --- | --- |
| 30 | Neuroradiology | 23 | 9 | MA |
| 31 | European journal of radiology | 14 | 3 | SR |
| 32 | Radiology | 26 | 10 | MA |
| 33 | Neuroradiology | 10 | 2 | SR |
| 34 | European journal of radiology | 23 | 6 | MA |
| 35 | Molecular imaging and biology | 23 | 7 | SR |
| 36 | JMRI | 10 | 2 | SR |
| 37 | Ultrasound in obstetrics & gynecology | 17 | 4 | SR |
| 38 | AJR | 24 | 7 | MA |
| 39 | European radiology | 22 | 9 | MA |
| 40 | Radiology | 18 | 5 | MA |
| 41 | Radiology | 25 | 9 | MA |
| 42 | European journal of radiology | 16 | 6 | SR |
| 43 | European journal of radiology | 27 | 10 | MA |
| 44 | European journal of radiology | 17 | 4 | SR |
| 45 | European journal of radiology | 27 | 10 | MA |
| 46 | European journal of radiology | 27 | 9 | MA |
| 47 | European radiology | 23 | 5 | MA |
| 48 | European radiology | 25 | 9 | MA |
| 49 | European radiology | 23 | 9 | SR |
| 50 | Radiology | 26 | 10 | MA |
| 51 | JMRI | 15 | 2 | MA |
| 52 | Ultrasound in obstetrics & gynecology | 21 | 7 | SR |
| 53 | Ultrasound in obstetrics & gynecology | 23 | 9 | SR |
| 54 | AJNR | 18 | 3 | SR |
| 55 | Radiology | 24 | 9 | MA |
| 56 | Ultrasound in obstetrics & gynecology | 15 | 2 | MA |
| 57 | European radiology | 26 | 9 | MA |
| 58 | European radiology | 25 | 9 | MA |
| 59 | Ultrasound in obstetrics & gynecology | 22 | 8 | MA |
| 60 | Radiology | 23 | 8 | MA |
| 61 | JMRI | 26 | 10 | MA |
| 62 | European journal of radiology | 26 | 10 | MA |
| 63 | European journal of radiology | 15 | 5 | SR |
| 64 | AJR | 26 | 9 | MA |
| 65 | Radiology | 23 | 9 | MA |
| 66 | European journal of radiology | 26 | 8 | MA |
| 67 | Radiology | 26 | 10 | MA |
| 68 | European radiology | 26 | 11 | MA |
| 69 | Ultrasound in obstetrics & gynecology | 26 | 10 | MA |
| 70 | Ultraschall in der Medizin | 16 | 5 | SR |
| 71 | Ultrasound in obstetrics & gynecology | 26 | 10 | MA |
| 72 | Radiology | 26 | 10 | MA |
| 73 | European radiology | 25 | 9 | MA |
| 74 | AJNR | 24 | 9 | MA |
| 75 | Radiology | 27 | 10 | MA |
| 76 | AJNR | 12 | 2 | SR |
| 77 | European journal of radiology | 22 | 8 | SR |
| 78 | European radiology | 8 | 1 | SR |
| 79 | European radiology | 25 | 9 | MA |
| 80 | Radiology | 25 | 8 | MA |
| 81 | European journal of radiology | 20 | 5 | SR |
| 82 | Radiology | 26 | 10 | MA |
| 83 | European journal of radiology | 26 | 10 | SR |
| 84 | JMRI | 25 | 9 | MA |
| 85 | Radiology | 26 | 10 | MA |
| 86 | Journal of cardiovascular magnetic resonance | 24 | 9 | MA |
| 87 | AJR. American journal of roentgenology | 26 | 10 | MA |
| 88 | Ultrasound in obstetrics & gynecology | 17 | 5 | MA |
| 89 | Radiology | 24 | 9 | MA |
| 90 | Ultrasound in obstetrics & gynecology | 21 | 7 | SR |
| 91 | European radiology | 23 | 8 | SR |
| 92 | AJNR | 21 | 7 | SR |
| 93 | Radiology | 23 | 8 | SR |
| 94 | European journal of radiology | 24 | 9 | MA |
| 95 | European journal of radiology | 23 | 9 | MA |
| 96 | AJNR | 23 | 8 | SR |
| 97 | Ultrasound in obstetrics & gynecology | 23 | 8 | MA |
| 98 | JMRI | 25 | 9 | MA |
| 99 | Radiology | 22 | 8 | SR |
| 100 | JMRI | 19 | 5 | SR |
| 101 | European radiology | 24 | 9 | SR |
| 102 | AJR | 17 | 3 | SR |
| 103 | European journal of radiology | 21 | 5 | SR |
| 104 | European radiology | 25 | 8 | MA |
| 105 | AJR | 23 | 7 | MA |
| 106 | European journal of radiology | 25 | 9 | MA |
| 107 | AJNR | 21 | 5 | SR |
| 108 | European radiology | 23 | 3 | SR |
| 109 | AJR | 21 | 3 | MA |
| 110 | European radiology | 23 | 8 | SR |
| 111 | Ultrasound in obstetrics & gynecology | 23 | 8 | MA |
| 112 | Radiology | 24 | 9 | SR |
| 113 | AJR | 19 | 8 | SR |
| 114 | Ultraschall in der Medizin | 22 | 9 | MA |
| 115 | European journal of radiology | 22 | 6 | SR |
| 116 | European radiology | 21 | 6 | MA |
| 117 | AJNR | 26 | 10 | MA |
| 118 | AJNR | 23 | 8 | SR |
| 119 | European radiology | 23 | 8 | MA |
| 120 | Ultrasound in obstetrics & gynecology | 22 | 8 | MA |
| 121 | Radiology | 26 | 11 | MA |
| 122 | Ultraschall in der Medizin | 9 | 2 | SR |
| 123 | AJR | 24 | 9 | MA |
| 124 | Radiology | 23 | 9 | SR |
| 125 | European radiology | 20 | 5 | MA |
| 126 | Neuroradiology | 17 | 6 | MA |
| 127 | European radiology | 24 | 9 | MA |
| 128 | AJNR | 17 | 2 | SR |
| 129 | Radiology | 24 | 9 | MA |
| 130 | AJNR | 16 | 5 | SR |
| 131 | European radiology | 24 | 9 | MA |
| 132 | Neuroradiology | 19 | 5 | SR |
| 133 | AJR | 22 | 9 | MA |
| 134 | European journal of radiology | 13 | 1 | SR |
| 135 | European journal of radiology | 20 | 5 | SR |
| 136 | European journal of radiology | 10 | 3 | SR |
| 137 | Radiology | 26 | 10 | MA |
| 138 | Ultrasound in obstetrics & gynecology | 22 | 7 | SR |
| 139 | Radiology | 25 | 9 | MA |
| 140 | Ultrasound in obstetrics & gynecology | 14 | 2 | SR |
| 141 | European journal of radiology | 11 | 3 | SR |
| 142 | Radiology | 25 | 7 | MA |
| 143 | European journal of radiology | 23 | 8 | SR |
| 144 | European radiology | 23 | 8 | MA |
| 145 | Radiology | 24 | 5 | SR |
| 146 | AJR | 20 | 6 | MA |
| 147 | AJNR | 15 | 1 | SR |
| 148 | Radiology | 24 | 9 | MA |
| 149 | Radiology | 26 | 9 | MA |
| 150 | Radiology | 26 | 10 | MA |
| 151 | Ultrasound in obstetrics & gynecology | 21 | 6 | MA |
| 152 | European journal of radiology | 21 | 6 | MA |
| 153 | Radiology | 26 | 10 | MA |
| 154 | Radiology | 26 | 10 | MA |
| 155 | Ultrasound in obstetrics & gynecology | 23 | 5 | MA |
| 156 | Ultrasound in obstetrics & gynecology | 23 | 8 | MA |
| 157 | Ultrasound in obstetrics & gynecology | 21 | 10 | SR |
| 158 | Ultrasound in obstetrics & gynecology | 23 | 8 | MA |

SR = Systematic Review, MA = Meta-analysis, AJR = American Journal of Roentgenology, JMRI = Journal of Magnetic Resonance Imaging, AJNR = American Journal of Neuroradiology
